# One‐Step Chemo‐, Regio‐ and Stereoselective Reduction of Ketosteroids to Hydroxysteroids over Zr‐Containing MOF‐808 Metal‐Organic Frameworks

**DOI:** 10.1002/chem.202100967

**Published:** 2021-06-08

**Authors:** H.‐H. Mautschke, F. X. Llabrés i Xamena

**Affiliations:** ^1^ Instituto de Tecnología Química Universitat Politècnica de València Consejo Superior de Investigaciones Científicas Avda. de los Naranjos s/n 46022 Valencia Spain

**Keywords:** heterogeneous catalysis, Meerwein-Ponndorf-Verley, metal-organic frameworks, steroids

## Abstract

Zr‐containing MOF‐808 is a very promising heterogeneous catalyst for the selective reduction of ketosteroids to the corresponding hydroxysteroids through a Meerwein‐Ponndorf‐Verley (MPV) reaction. Interestingly, the process leads to the diastereoselective synthesis of elusive 17α‐hydroxy derivatives in one step, whereas most chemical and biological transformations produce the 17β‐OH compounds, or they require several additional steps to convert 17β‐OH into 17α‐OH by inverting the configuration of the 17 center. Moreover, MOF‐808 is found to be stable and reusable; it is also chemoselective (only keto groups are reduced, even in the presence of other reducible groups such as C=C bonds) and regioselective (in 3,17‐diketosteroids only the keto group in position 17 is reduced, while the 3‐keto group remains almost intact). The kinetic rate constant and thermodynamic parameters of estrone reduction to estradiol have been obtained by a detailed temperature‐dependent kinetic analysis. The results evidence a major contribution of the entropic term, thus suggesting that the diastereoselectivity of the process is controlled by the confinement of the reaction inside the MOF cavities, where the Zr^4+^ active sites are located.

## Graphical abstract

**Zr‐containing MOF‐808 is a stable and reusable catalyst** for the chemo‐, regio‐ and diastereoselective reduction of 17‐ketosteroids to elusive 17α‐hydroxysteroids compounds, as shown for the synthesis of 17α‐estradiol, androstan‐3β,17α‐diol, and epitestosterone. The process was carried out in a single step and without using toxic and/or expensive reagents. Temperature‐dependent kinetic analysis of estrone reduction revealed that the diastereoselectivity to 17α‐estradiol originates from the confinement of the reaction inside the MOF cavities in which the Zr^4+^ active sites are located.

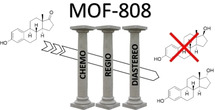

## Introduction

**Scheme 1:**
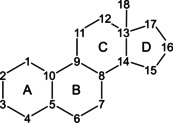
Steroids skeleton and conventional atom and ring labeling.

Steroids are a large group of chemical substances sharing a 17‐carbon atom skeleton composed of four fused rings (three six‐membered rings and one five‐membered ring), conventionally denoted A–D, as shown in Scheme 1.^[1]^ Steroid compounds vary from each other in the type of groups attached to this skeleton, their position, and configuration. Small modifications in the structure of these steroids produce significant differences in their biological activity. Therefore, the development of novel routes to modify existing steroids in a controlled way to improve their activity or to prepare new steroid derivatives with potential pharmacologic activity is an area of active research. In this sense, chemo‐, regio‐ and stereoselective (bio)transformation of steroids using highly selective catalysts or microorganisms is crucial.

Within the large family of steroids, ketosteroids (also denoted oxosteroids) are steroids in which at least one hydrogen atom of the steroid nucleus has been replaced by a keto group (C=O). Many endogenous hormones are ketosteroids, as well as their synthetic analogs used in medicine, such as glucocorticoids and corticoids in general.^[2]^ Meanwhile, hydroxysteroids are also relevant compounds in the pharmaceutical industry, and in particular those containing a hydroxyl group attached to C17, such as estradiol, androstanediol or testosterone/epitestosterone. One possible route to prepare these hydroxysteroids is by a direct stereoselective reduction of the corresponding ketosteroid, which can be carried out by using either a variety of microorganisms^[3]^ or with chemical reducing agents.

In general, biotransformation has several advantages over chemical routes, including regio‐, stereo‐ and enantioselectivity, thus allowing the preparation of chiral products. From an economic point of view, biotransformations can also be cheaper than chemical methods. However, in many cases, the reduction of ketosteroids to hydroxysteroids by microorganisms is accompanied by unwanted side reactions, such as C=C bond reduction or hydroxylation at other positions of the steroid skeleton,[^4^, ^5^, ^6^] that decrease the final yield of the target hydroxysteroids and complicate their purification and isolation. Additional complications may arise in biocatalytic processes due to the low solubility in aqueous medium of steroids in general, or related to the regeneration of the cofactors (NADH or NADPH) used as the source of hydride ions in the process.

**Scheme 2:**
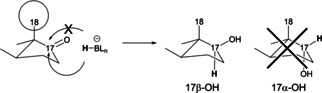
Reduction of 17‐ketosteroids by most chemical reducing compounds yields 17β‐OH compounds due to the steric hindrance of the 18‐methyl group.

Besides biocatalytic methods, reduction of ketosteroids can be also achieved by using chemical reducing agents, such as NaBH_4_, Zn(BH_4_)_2_ or LiAlH_4_, though these methods present their own limitations as well. For instance, reduction of 17‐ketosteroids with NaBH_4_ produces exclusively 17β‐OH isomers due to the steric hindrance of the 18‐methyl group that blocks hydride attack from the upper face (Scheme 2). Therefore, when the product of interest is the 17α‐OH isomer, most efforts have been focused on inverting the configuration of 17β‐OH through Mitsunobu reactions,[^7^, ^8^] or by the formation and displacement of sulfonyl ester derivatives.[^9^, ^10^, ^11^] However, both methods introduce further synthetic steps, including protection/deprotection reactions using expensive or non‐commercial reagents, and complicating product isolation and purification. As a result, the final yield obtained of the target 17α‐OH is usually very low.

Besides stereoselectivity, regioselectivity can also be an issue when chemical reducing agents are used for the reduction of steroids bearing more than one keto group. For instance, 3‐ketosteroids are in general more reactive than 17‐or 20‐ketosteroids.^[12]^ Therefore, the preparation of 17‐OH products can be challenging when more than one keto groups are present in the starting ketosteroid, so that mixtures of various mono‐ and polyhydroxylated products can be obtained. Finally, also chemoselectivity of the reaction must be taken into account when working with ketosteroids containing other reducible groups (such as C=C bonds) besides the target carbonyl group.

Zirconium trimesate MOF‐808 is a metal‐organic framework (see structure in Figure S1 in the Supporting Information) that was first described in 2014 by Furukawa et al.^[13]^ This compound has recognized catalytic activity for the Meerwein‐Ponndorf‐Verley (MPV) reduction of various carbonyl compounds.[^14^, ^15^, ^16^] In this sense, we have shown^[16]^ that pristine and defect‐engineered MOF‐808 are more active for this reaction than the archetypal zirconium terephthalate, UiO‐66.^[17]^ This was attributed to a high availability of coordinatively unsaturated Zr^4+^ sites (*cus*) in MOF‐808 upon removal of formate ions used during the synthesis. Meanwhile, *cus* in UiO‐66 are only located at missing‐linker defect sites and are thus much less abundant.^[18]^ A further advantage of MOF‐808 compared to UiO‐66 is its wider pore structure (pore apertures are 14 Å in MOF‐808 and ∼6 Å in UiO‐66), which allows converting bulkier substrates. We showed this by comparing the MPV reduction of estrone, with approximate dimensions: 11.2×6.2×4.2 Å, using either MOF‐808 or UiO‐66 as catalyst. Our results showed that UiO‐66 was basically inactive for this reaction, while MOF‐808 produced the expected hydroxylated derivative quantitatively. Interestingly, MPV reduction of estrone with isopropanol over MOF‐808 yielded the elusive 17α‐estradiol with moderate diastereoselectivity. Although the diastereoselectivity attained in that work was still far from optimum, it is worth mentioning that 17α‐hydroxy‐estradiol was obtained directly from estrone in a single reaction step, using only isopropanol as the sole reagent and avoiding any additional protection/deprotection steps (as required, for instance, in Mitsunobu type processes[^7^, ^8^]).

Encouraged by these promising results, we wanted to explore in more detail the diastereoselective reduction of estrone over MOF‐808 materials. We have extended our studies to the synthesis of other challenging 17α‐hydroxysteroids of interest from the corresponding 17‐ketosteroids. Selection of these compounds allowed us to further address other relevant aspects of the catalytic process, such as regio‐ and chemoselectivity of the MPV reaction as well as catalyst stability and reusability.

## Results and Discussion

### Diastereoselective reduction of estrone

**Scheme 3:**
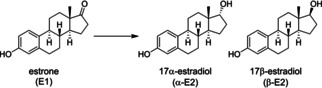
Reduction of E1 yields a mixture of α‐E2 and β‐E2 diastereoisomers.

The reduction of estrone (hereafter E1) at the 17 position can yield a mixture of two estradiols, as shown in Scheme 3. These two alcohols are diastereoisomers and are termed 17α‐ and 17β‐estradiol (hereafter α‐E2 and β‐E2 for short), depending on the configuration of the 17‐OH group relative to the 18‐methyl group.

As shown in Table 1 (entry 1), when reduction of E1 was carried out over MOF‐808 in isopropanol (*i*PrOH) at 393 K, the reaction was almost complete after 8 h (97 % conversion). The corresponding turnover frequency (TOF), calculated at short reaction time, was 2.44 h^−1^. As compared to *i*PrOH, the reaction was slightly slower when butan‐2‐ol (2‐BuOH) was used (TOF=1.33 h^−1^), but it eventually reached almost full conversion (91 %) after 8 h, and complete conversion after 24 h of reaction (entry 2). The reaction also proceeded smoothly with pentan‐2‐ol (2‐PentOH) and 1‐phenyl ethanol (entries 3 and 4). In all cases, the reduction of estrone produced only a mixture of α‐E2 and β‐E2, and no other by‐products were detected (100 % selectivity).

**Table 1:** Summary of the catalytic results for the MPV reduction of estrone over various catalysts.^[a]^

	Catalyst	Alcohol	Conv. time^[b]^	TOF^[c]^ [h^−1^]	*dr* ^[d]^
1	MOF‐808	*i*PrOH	97 % (8 h) >99 % (24 h)	2.44	60 : 40
2	MOF‐808	2‐BuOH	91 % (8 h) >99 % (24 h)	1.33	87 : 13
3	MOF‐808	2‐PentOH	92 % (8 h)	2.11	73 : 28
4	MOF‐808	1‐phenylethanol	97 % (8 h)	2.77	60 : 40
5	ZrO_2_	2‐BuOH	2 % (24 h)	–	44 : 56
6	ZrCl_4_	2‐BuOH	13 % (24 h)	–	50 : 50
7	Zr‐beta^[e]^	2‐BuOH	2 % (24 h)	–	65 : 35
8	Zr‐MCM‐41^[e]^	2‐BuOH	2 % (24 h)	–	52 : 48
9	Zr(*i*PrO)_4 ⋅_ *i*PrOH	2‐BuOH	2 % (24 h)	–	50 : 50
10	Al(*i*PrO)_3_	2‐BuOH	11 % (24 h)	–	34 : 66
11	NaBH_4_^*f*^		99 %	–	3 : 97

[a] Reaction conditions: 20 mg of estrone (0.08 mmol), alcohol (ca. 16 equiv) and Zr‐catalyst (5 mg, ca. 18 mol% Zr), 393 K. [b] Conversion. Determined by GC. Estradiols were the only products detected. [c] Turnover frequency. Moles of estrone converted per mol of Zr and per hour of reaction. [d] α‐E2:β‐E2 diastereomeric ratio, calculated from the ^1^H NMR spectra of the reaction filtrates (for the exact procedure used, see the Supporting Information). [e] See the Supporting Information for a detailed description of these catalysts. [f] The procedure used for estrone reduction with NaBH_4_ is described in the Experimental Section.

The results obtained with MOF‐808 are in sharp contrast with the little or no catalytic activity observed under the same reaction conditions with other Zr‐containing compounds (entries 5–8), or using well‐known MPV catalysts, such as Zr‐ and Al‐isopropoxides (entries 9 and 10).

The diastereomeric ratio of the two alcohols formed, α‐E2:β‐E2, was determined at the end of the reaction from the corresponding ^1^H NMR spectra of the reaction filtrate. The procedure used is detailed in the Supporting Information. The results obtained are also included in the last column in Table 1.

As we reported in our previous work, the use of MOF‐808 as catalyst for the MPV reduction of estrone produced a moderate amount of α‐E2, while most available biotransformation methods and chemical routes produce almost exclusively the β‐E2 isomer (see entry 11 in Table 1 for the results obtained with NaBH_4_). This is a very motivating result from the practical point given the current interest in α‐E2 for its anti‐oxidative and anti‐inflammatory properties and as a potent ligand for a membrane‐associated ER‐X receptor and the activation of MAPK/ERK.^[19]^ α‐E2 is of pharmaceutical interest for treating degenerative diseases, such as Alzheimer, Parkinson, Friedreich's ataxia or stroke, with much less secondary effects than the β‐E2 isomer.[^20^, ^21^, ^22^, ^23^] Therefore, the development of new synthetic (catalytic) routes for the preparation of this compound from cheap starting compounds, such as estrone, has an obvious interest.

In our previous work,^[16]^ a ca. 40 : 60 α‐E2: β‐E2 *dr* was achieved when *i*PrOH was used at 353 K. Thus, although α‐E2 was still the minor product of the reaction, the amount obtained was still considerably higher than in previous reports on the synthesis of similar 17α‐OH compounds.[^12^, ^24^, ^25^] In the present case, the results obtained with *i*PrOH at 393 K were significantly better: 60 % selectivity to α‐E2 (entry 1), thus becoming the major product of the reaction. Since the diastereoselectivity attained in the MPV reduction of estrone does not seem to depend on the reaction temperature (see below), we attribute the higher selectivity to α‐E2 attained in the present work to improvements in the MOF‐808 preparation and activation procedure (viz. formate removal) compared to our previous study.

Still better results were obtained when 2‐BuOH was used as a solvent and reducing alcohol, attaining an impressive 87 : 13 *dr* at 91 % conversion after 8 h of reaction (entry 2). When the size of the secondary alcohol used was further increased (viz., 2‐PentOH and 1‐phenyl ethanol), the *dr* decreased to 73 : 28 and 60 : 40, respectively, thus evidencing that 2‐BuOH provided the optimum diastereoselectivity among the alcohols tested.

**Figure 1:**
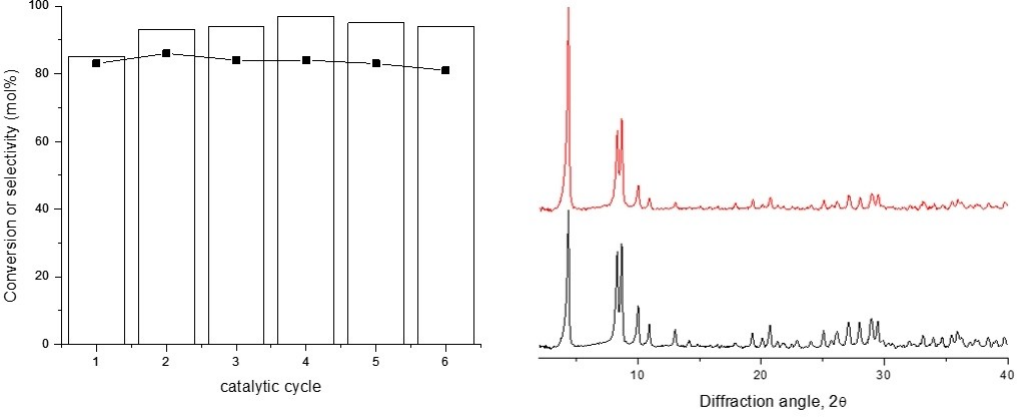
Reusability of MOF‐808 for the MPV reduction of estrone with 2‐BuOH at 393 K. Left: Estrone conversion (□) and selectivity to α‐E2 (▪). Right: X‐ray powder diffraction pattern of fresh MOF‐808 (black) and MOF‐808 recovered after 6 catalytic cycles (red).

We thus considered the reaction with 2‐BuOH at 393 K to evaluate the stability and reusability of MOF‐808. According to the XRD characterization of the catalyst recovered after the MPV reaction, the solid was found to be stable under the reaction conditions used, and both the catalytic activity and diastereoselectivity to α‐E2 was maintained for at least 6 catalytic cycles, as shown in Figure 1. Meanwhile, ICP analysis of the filtrate after the reaction revealed a minimal Zr^4+^ leaching (<1 % of the total zirconium used) from the catalyst to the reaction medium.

### Effect of the reaction temperature

**Figure 2:**
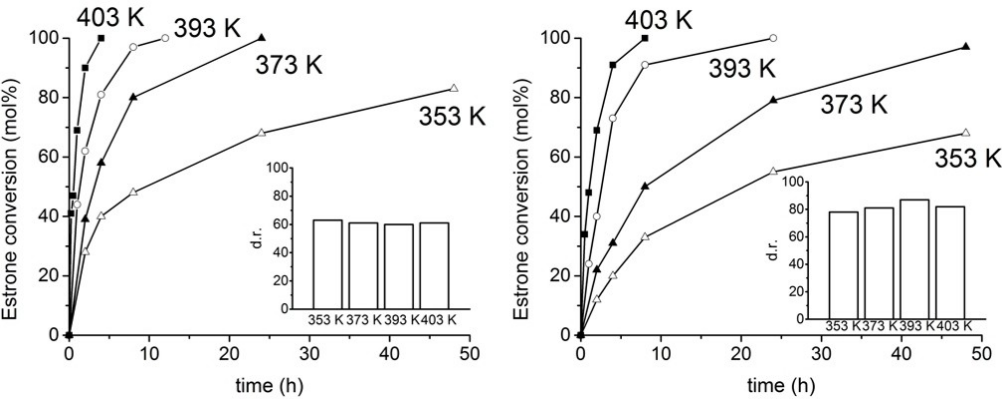
Time‐conversion plots of MPV reduction of estrone by using MOF‐808 with isopropanol (left) or butan‐2‐ol (right) over the temperature range 353–403 K. In each plot, the inset shows the diastereomeric ratio of the reaction, *dr* (α‐E2: β‐E2).

The effect of reaction temperature on the product distribution was assessed with both *i*PrOH and 2‐BuOH in the temperature range from 353 to 403 K. Figure 2 shows the time‐conversion plots obtained and the corresponding α‐E2: β‐E2 *dr* measured at the end of the reaction (white bars in the insets).

Upon raising the reaction temperature from 353 to 403 K, a sharp and progressive increase of the reaction rate was observed for both *i*PrOH and 2‐BuOH. Thus, in the case of *i*PrOH, full estrone conversion was attained after only 4 h of reaction at 403 K, while up to 48 h were needed to reach 83 % conversion at 353 K. Thus, calculated TOFs increased from 0.78 to 9.11 h^−1^ in the 353–403 K range. The same holds for 2‐BuOH, for which TOF increased from 0.33 to 3.78 h^−1^ from 353 to 403 K.

Meanwhile, the diastereoselectivity of the reaction remained almost unchanged within the whole temperature range and depended only on the alcohol used (see insets in Figure 2). This lack of kinetic or thermodynamic control is in agreement with the reaction taking place inside the MOF cavities (where the Zr^4+^ are located) and being affected mainly by cavity confinement effects during the formation of the transition state (see below).

### Kinetic analysis

**Figure 3:**
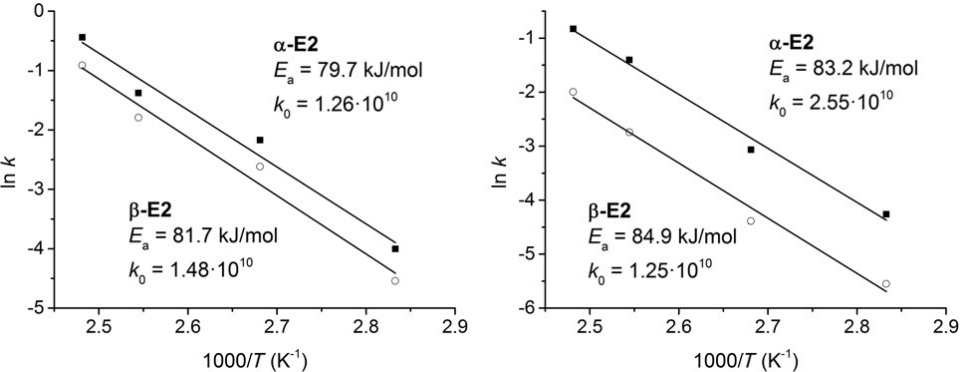
Arrhenius plots for the conversion of estrone to α‐E2 and β‐E2 in *i*PrOH (left) and 2‐BuOH (right), with calculated apparent activation energies (*E*_a_) and pre‐exponential factors (*k*_0_) for each compound.

Kinetic rate constants of α‐E2 and β‐E2 formation (hereafter *k*_α_ and *k*_β_) were calculated for both *i*PrOH and 2‐BuOH in the 353–403 K temperature range, as detailed in the Supporting Information. Apparent activation energies (*E*_a_) for the formation of α‐E2 and β‐E2 were calculated from the slopes of the corresponding Arrhenius plots shown in Figure 3, while the intercept with the *y*‐axis was used to calculate the pre‐exponential factors, *k*_0_, which are correlated with entropic factors. To assess the goodness of the fit, the values of *E*_a_ and *k*_0_ obtained for each solvent were used to plot the theoretical evolution of the reaction rate constant, *k*, as a function of the temperature according to the formula *k*=*k*_0_ exp(−*E*_a_/RT) and compared with the experimental data (Figure S6).

The calculated activation energies are in general lower for *i*PrOH than for 2‐BuOH, reflecting the faster E1 conversion observed for *i*PrOH (Figure 2). For both *i*PrOH and 2‐BuOH, the formation of α‐E2 and β‐E2 have very similar apparent activation energies, being only slightly lower for α‐E2 than for β‐E2: 79.7 and 81.7 kJ/mol for *i*PrOH, and 83.2 and 84.9 kJ/mol for 2‐BuOH. Meanwhile, the pre‐exponential factors are in all cases comparable, all of them of the same order of magnitude.

Given the small differences observed in both *E*_a_ and *k*_0_ values, it is difficult at this point to determine what are the main factors governing the higher α‐E2 diastereoselectivity obtained with 2‐BuOH compared to *i*PrOH, or why the selectivity does not seem to be affected by the reaction temperature. Moreover, if only the differences in *E*_a_ between the two isomers (Δ*E*_a_) are considered, one would expect a slightly higher selectivity towards α‐E2 in *i*PrOH than in 2‐BuOH (since Δ*E*_a_=2 kJ/mol for *i*PrOH, and Δ*E*_a_=1.7 kJ/mol for 2‐BuOH). This obviously does not correspond to the *dr* observed for this reaction. It is thus evident that entropic contributions must play a relevant role in determining the observed dependence of the reaction diastereoselectivity on the alcohol used, as we will show below.

**Scheme 4:**
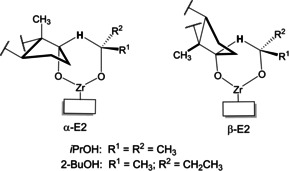
Proposed structures of the two possible transition states for the MPV reduction of estrone leading to α‐E2 and β‐E2 over MOF‐808, with either *i*PrOH or 2‐BuOH.

While the Arrhenius equation is an empirical model, the Eyring‐Polanyi equation, derived from transition state theory, can be used to calculate directly the values of the entropy (Δ*S* ^≠^), enthalpy (Δ*H* ^≠^), and Gibbs free energy (Δ*G* ^≠^) of activation for both α‐E2 and β‐E2 and for the two alcohols used (Figure S7 and Table 2). Here, Δ*G* ^≠^, Δ*H* ^≠^, and Δ*S* ^≠^ represent the changes in energy upon forming an activated transition state complex between estrone, the secondary alcohol, and the Zr^4+^ active site of MOF‐808. In this sense, we recall here that the generally accepted mechanism for the MPV reduction of ketones over Lewis acid catalysts assumes the simultaneous adsorption of the alcohol and the ketone on the active site, involving a six‐membered cyclic transition state,^[26]^ which in the case of estrone should have the structure shown in Scheme 4.

**Table 2:** Enthalpy, entropy and Gibbs free activation energy for α‐E2 and β‐E2 by using MOF‐808 over *i*PrOH and 2‐BuOH. The bottom part reports the differential activation thermodynamic parameters, calculated as the difference between the transition states leading to α‐E2 and β‐E2 (α–β) in terms of Gibbs free energy (ΔΔ*G* ^≠^), and breakdown into enthalpic (ΔΔ*H* ^≠^) and entropic contributions (−*T*ΔΔS^≠^).

		Δ*S* ^≠^ [J mol^−1^K^−1^]	−*T*Δ*S* ^≠ [a]^ [kJ mol^−1^]	Δ*H* ^≠^ [kJ mol^−1^]	Δ*G* ^≠ [a]^ [kJ mol^−1^]
*i*PrOH	α‐E2	−61.8	24.3	76.6	100.9
	β‐E2	−60.5	23.8	78.5	102.3
2‐BuOH	α‐E2	−56.0	22.0	80.0	102.0
	β‐E2	−61.9	24.3	81.8	106.1
		ΔΔ*S* ^≠^ [J mol^−1^ K^−1^]	−*T*ΔΔ*S* ^≠ [a]^ [kJ mol^−1^]	ΔΔ*H* ^≠^ [kJ mol^−1^]	ΔΔ*G* ^≠ [a]^ [kJ mol^−1^]
α‐β	*i*PrOH	−1.3	+0.5	−1.9	−1.4
	2‐BuOH	+5.9	−2.3	−1.8	−4.1

[a] Calculated at 393 K.

**Figure 4:**
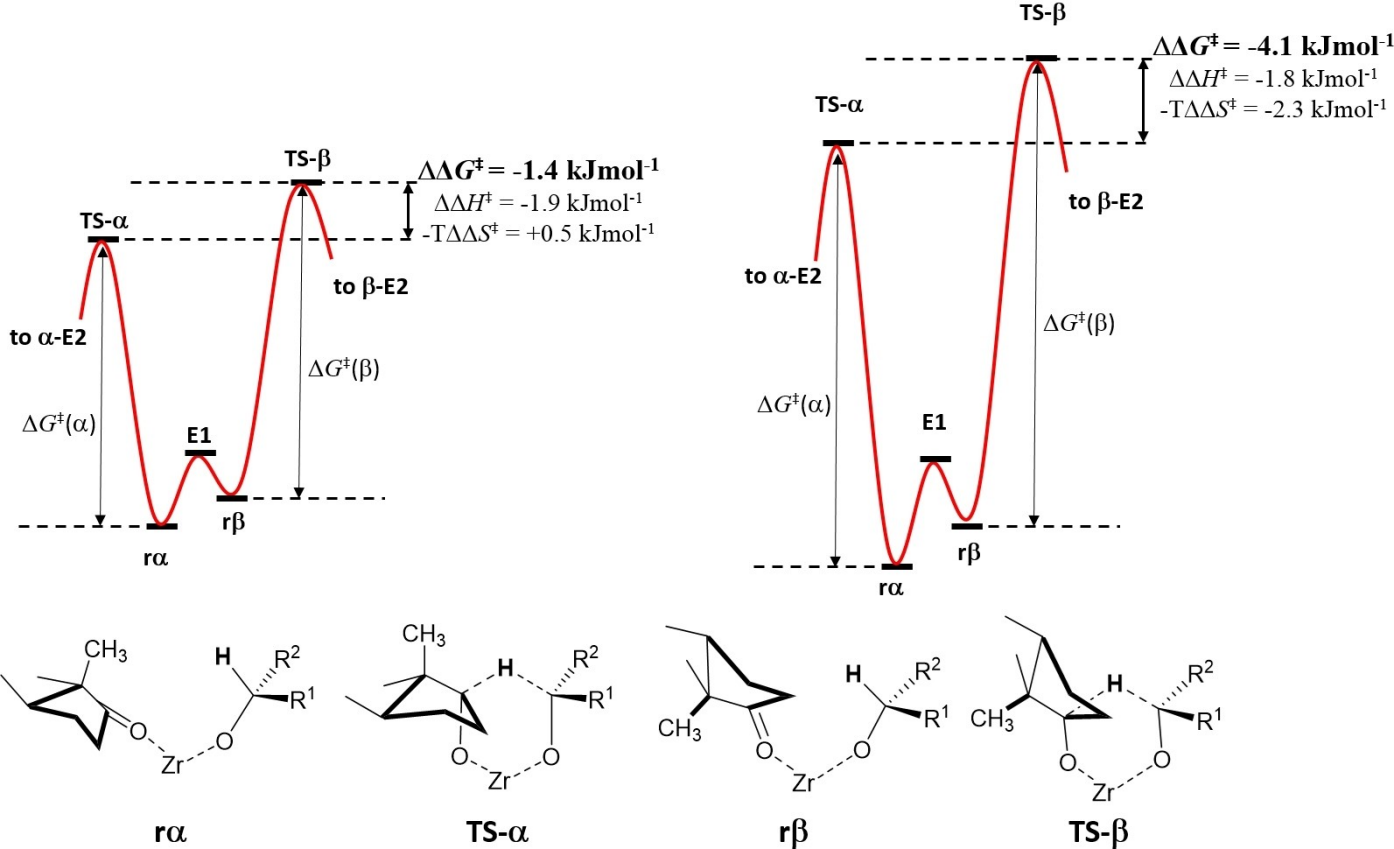
Schematic energy diagram (not to scale) showing the differences in energies between the two activated complexes leading to α‐E2 and β‐E2 in *i*PrOH (left) and 2‐BuOH (right). The bottom part show simple snapshots representing the most relevant structures formed in the course of the reaction.

For a given solvent, we can also evaluate differential activation parameters (ΔΔ*G* ^≠^, ΔΔ*H* ^≠^ and ΔΔ*S* ^≠^), which correspond to the difference between the transition states leading to α‐E2 and β‐E2. These differential parameters are also reported in the bottom part of Table 2 (α–β). To help interpret the meaning of these parameters, Figure 4 shows a schematic representation (not to scale) of the energy profile of the reaction with *i*PrOH and 2‐BuOH, as well as simple snapshots representing the most relevant structures formed in the course of the reaction.

As can be seen in Table 2, when the MPV reaction is carried out in 2‐BuOH at 393 K, the formation of the activated complex leading to α‐E2 is preferred over the other transition state by 4.1 kJ mol^−1^ (ΔΔ*G* ^≠^ in bottom part in Table 2, see also Figure 4), while this difference is only 1.4 kJ mol^−1^ in the case of *i*PrOH. Thus, ΔΔ*G* ^≠^ values in Table 2 directly reflect the higher *dr* obtained for 2‐BuOH than for *i*PrOH: ca. 82 % and 61 % in the whole 353–403 K temperature range (see insets in Figure 2).

For the reaction in 2‐BuOH, the entropy loss upon the formation of the transition state is lower for α‐E2 than for β‐E2 (−56.0 vs. −61.9 J mol^−1^ K^−1^). Therefore, both the differential enthalpic (ΔΔ*H* ^≠^=−1.8 kJ/mol) and entropic contributions (−*T*ΔΔ*S* ^≠^=−2.3 kJ/mol) have the same sign, and both contribute to lowering the energy of the transition state of α‐E2 compared to β‐E2. In contrast, for the reaction in *i*PrOH, the differential enthalpic and entropic terms have opposite signs (−1.9 and +0.5 kJ/mol, respectively), partially counteracting each other. As a consequence, the energy difference between both transition states is much lower in *i*PrOH.

Taking the above data together, our results suggest that the space available inside the MOF cavities can accommodate better the transition state TS‐α than TS‐β, and this difference is higher with 2‐BuOH than with *i*PrOH. This is not surprising if we consider that 2‐BuOH (or *i*PrOH) directly participate in the formation of the activated complex (Scheme 4).

Therefore, in the case of 2‐BuOH, the important contribution from the entropic term on the total energy difference between the two possible transition states strongly indicates that confinement effects and steric hindrance inside the MOF cavity are the main reasons determining the preferred reaction pathway and the observed diastereoselectivity. Accordingly, the selectivity to α‐E2 is much lower for the reaction with *i*PrOH, for which the entropic term partially neutralizes the enthalpic contribution, and the energy difference is therefore lower.

### Diastereoselective reduction of 5α‐androstan‐3β‐ol,17‐one (epiandrosterone)

**Scheme 5:**
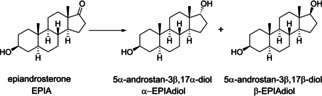
Reduction of epiandrosterone (EPIA) can yield a mixture of androstanediols (α‐EPIAdiol and β‐EPIAdiol).

Given the excellent diastereoselectivity attained with MOF‐808 for the reduction of estrone to 17α‐estradiol, we wanted to extend the study to the reduction of other ketosteroids. In this case, we considered the reduction of epiandrosterone (hereafter EPIA), which also bears a keto group in position 17. Similar to estrone, EPIA reduction can yield two diastereoisomers, 5α‐androstan‐3β,17α‐diol (α‐EPIAdiol) and 5α‐androstan‐3β,17β‐diol (β‐EPIAdiol), depending on the relative conformation of the 17‐OH group with respect to the 18‐CH_3_ group (Scheme 5):

Analogous to the case of estrone reduction, the presence of the CH_3_ group in position 18 introduces a strong steric hindrance to the hydride addition to the carbonyl from the upper face, so β‐EPIAdiol is usually the only product obtained in most synthetic routes. Thus, the preparation of the elusive α‐EPIAdiol compound is a challenging synthetic process. Nevertheless, α‐EPIAdiol can be an interesting target for the treatment of cancer, due to anti‐androgenic properties, and in the treatment of osteoporosis,[^27^, ^28^] so new selective synthetic routes are also sought for this compound.

**Figure 5:**
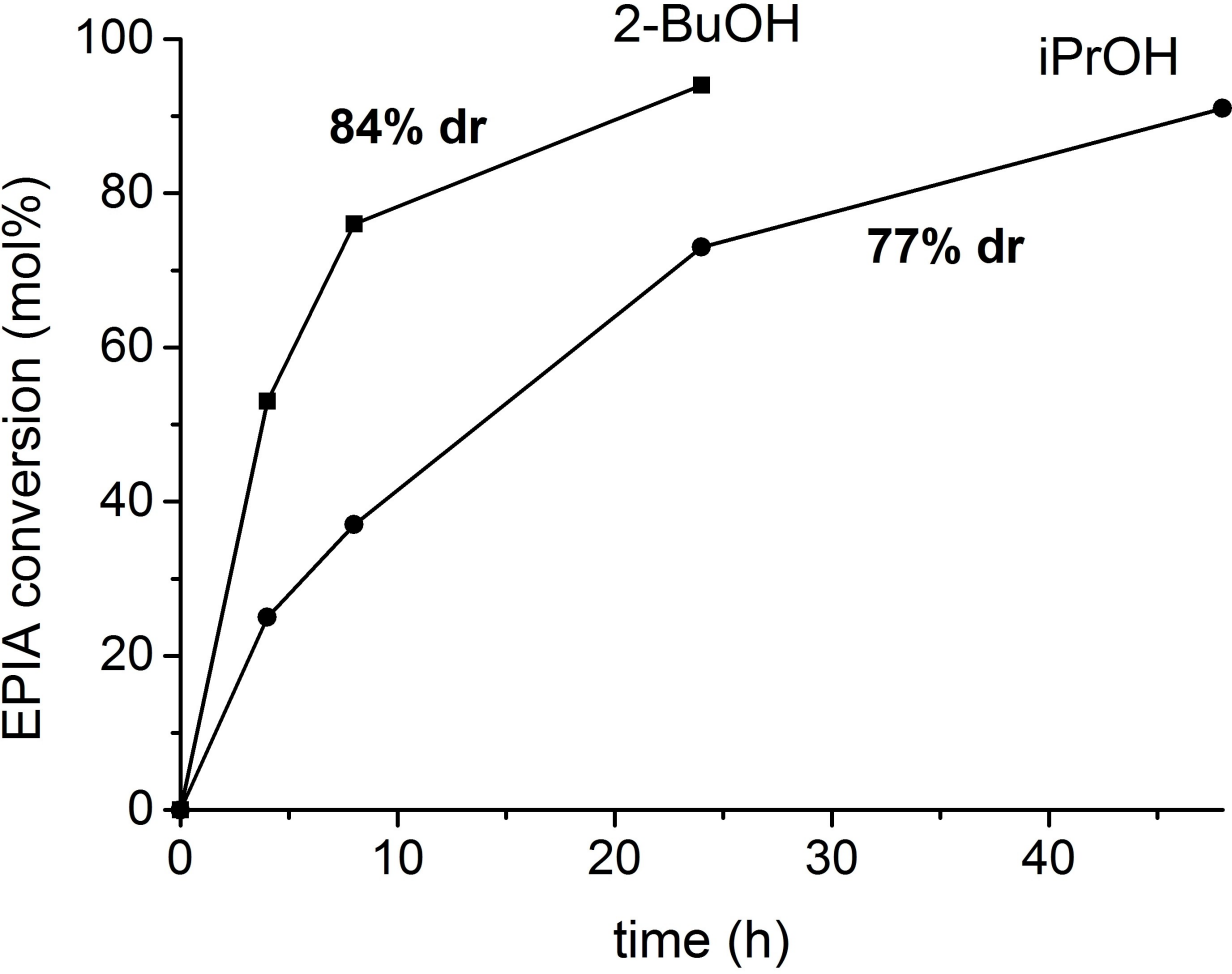
MPV reduction of epiandrosterone (EPIA) to androstanediols using *i*PrOH and 2‐BuOH. The obtained diastereoselectivity (*dr*) to α‐EPIAdiol is indicated. *dr* was calculated by ^1^H NMR of the filtrate at the end of the reaction (see the Supporting Information). Reaction conditions: 23 mg of EPIA (0.08 mmol), alcohol (ca. 16 equiv) and Zr catalyst (5 mg, ca. 18 mol% Zr), 393 K.

To our delight, MPV reduction of EPIA using MOF‐808 as catalyst produces α‐EPIAdiol as the main product with excellent diastereoselectivities, depending on the secondary alcohol used: 77 % *dr* and 84 % *dr* in the case of *i*PrOH and 2‐BuOH, respectively, as shown in Figure 5. In both cases, androstanediols were the only products observed, and the catalyst was found to be stable and reusable.

Together with estrone, the results obtained for the MPV reduction of EPIA confirm the general applicability of MOF‐808 as a stable and reusable catalyst for the diastereoselective reduction of 17‐ketosteroids to the corresponding 17α‐hydroxysteroids. Thus, MPV reduction of ketosteroids over MOF‐808 represents a very attractive and reliable one‐step catalytic alternative for the existing stoichiometric reactions using hydride transfer compounds. As mentioned in the introduction, most existing chemical methods usually yield 17β‐OH compounds, so preparation of 17α‐OH isomers require several synthetic steps and they use toxic and/or expensive reagents, leading to very low final yields of the target compound.

### Regio‐ and diastereoselective reduction of androstenedione

To increase further the scope of MOF‐808 as a catalyst for the MPV reduction of ketosteroids, it would be highly desirable to develop a catalyst not only diastereoselective but also chemo‐ and regioselective. It is well‐known that MPV is, in general, a chemoselective process, which only transforms carbonyl groups whilst leaving other easily reducible functional groups intact, as in the case of unsaturated ketones.^[29]^ However, a more demanding situation is found when the substrate contains more than one carbonyl group that, in principle, can be reduced, leading to a mixture of mono‐ and polyhydroxylated compounds. In this case, the regioselectivity of the reduction process may become an issue.

Δ^4^‐Androstene‐3,7‐dione (androstenedione, hereafter A4) contains a C=C bond in position 4 and two keto groups in positions 3 and 17, so we considered it as an appropriate substrate to assess both chemo‐ and regioselectivity of the MPV reduction using MOF‐808 catalyst.

**Scheme 6:**
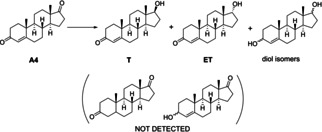
MPV reduction of androstenedione (A4) over MOF‐808 yields testosterone (T), epitestosterone (ET) and a mixture of diol isomers with different local configurations of the OH groups at positions 3 and 17.

**Figure 6:**
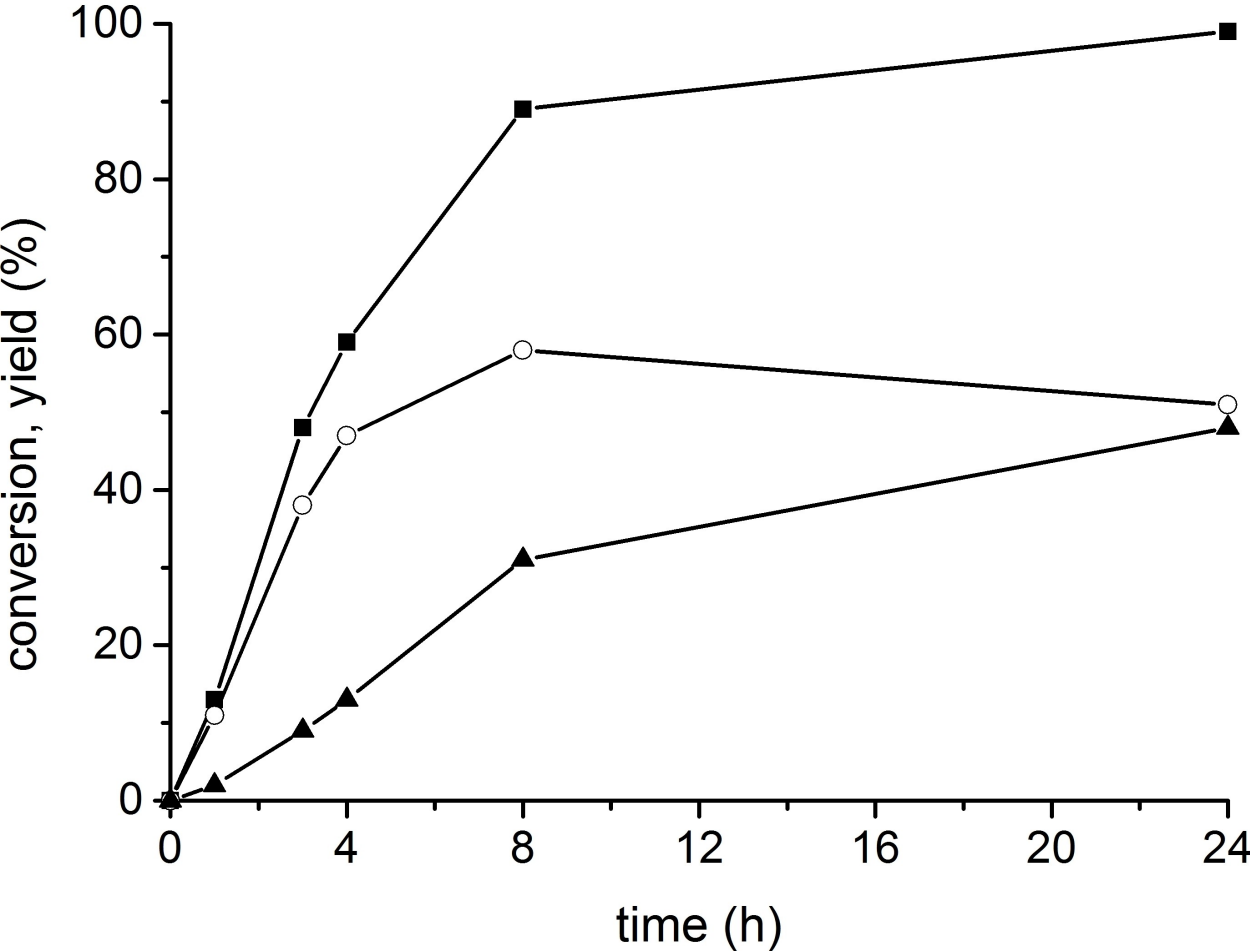
▪: A4 conversion, ○: yield of mono‐hydroxylated (T+ET), and ▴: yield of dihydroxylated products obtained over MOF‐808 with 2‐BuOH at 353 K. Reaction conditions: 10 mg of A4 (0.035 mmol), 1 mL 2‐BuOH (ca. 30 equiv) and 10 mg MOF‐808.

In our hands, upon reduction of A4 over MOF‐808 with either *i*PrOH or 2‐BuOH, we did not observe the formation of products coming from the reduction of the C=C bond (100 % chemoselectivity) or the mono‐reduction of the carbonyl group in position 3 (see the Supporting Information for more details on product identification by ^1^H and ^13^C NMR). Therefore, the only products detected were testosterone (T) and epitestosterone (E) from the reduction of the 17 keto group, and a mixture of diol isomers coming from the reduction of both keto groups, as shown in Scheme 6:

Figure 6 shows the time‐conversion plots and product distribution obtained with 2‐BuOH at 353 K; *i*PrOH afforded very similar results (Figure S8).

According to the time evolution of products shown in Figure 6, A4 reduction at the 17 position occurs in the first place, yielding a mixture of mono‐hydroxylated T and ET as primary products, followed afterwards by the reduction of the keto group also in position 3, to form the corresponding 3,17‐diols, at longer reaction times. Thus, the amount of 17‐mono‐hydroxylated products increases during the first 8 h of reaction, reaching a maximum yield of 58 %, and then starts to decrease gradually as more diols are formed.

Identification of the products using ^13^C and ^1^H NMR spectroscopy (see the Supporting Information) revealed that the amount of T formed was very low, close to the detection limits of the technique, and in any case, less than 5 % referred to ET. Therefore, as in the case of estrone and epiandrosterone discussed above, MOF‐808 yielded the corresponding 17α‐OH compound (viz, epitestosterone) with excellent diastereoselectivity over the 17β‐compound, higher than 95 %. This result is also interesting from the practical point of view, as ET has applications in the treatment of prostate cancer, due to its inhibitory effects of 5α‐reductase,^[30]^ and it has agonistic effects on a prostate‐specific G‐protein receptor (PSGR/OR51E2).^[31]^

**Figure 7:**
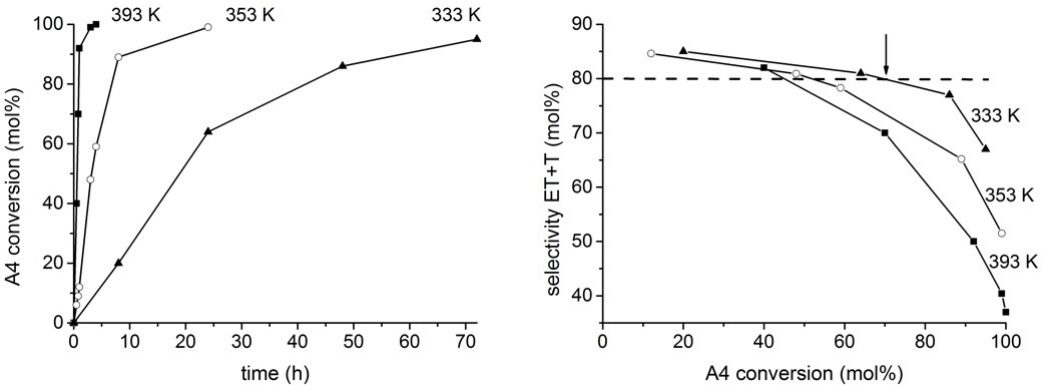
Left: A4 conversion over MOF‐808 in 2‐BuOH at the indicated temperature. Right: Selectivity to monohydroxylated products, ET+T, as a function of the A4 conversion and temperature.

In an attempt to improve the selectivity to mono‐hydroxylated products (T and ET) and minimize the formation of diols, the reaction temperature was varied over the 333–393 K range. Figure 7 shows the A4 conversion curves and corresponding selectivity‐conversion plots obtained as a function of the temperature. The complete kinetic data is given in Table S1.

As seen in Figure 7, the maximum selectivity to monohydroxylated products is around 85 % at 10–20 % conversion, but this value decreases gradually at higher levels of conversion, dropping faster as the reaction temperature increases. Therefore, increasing the reaction temperature has a clear detrimental effect on the selectivity to mono‐hydroxylated products. Nevertheless, our data show that if the reaction temperature is kept at 333 K, it is possible to attain a level of A4 conversion as high as 70–80 % after 30–35 h, while maintaining a selectivity to monohydroxylated compounds above 80 % (arrow in Figure 7) and a diastereoselectivity >95 % to ET, which is considered an excellent result.

Overall, our study has revealed that MOF‐808 affords a very good selectivity for the reduction of A4 to ET, as: i) the C=C bond in position 4 remains intact under the reaction conditions used (100 % chemoselectivity); ii) no traces of 3‐hydroxy‐17‐ketosteroids have been detected, so the 17‐carbonyl group is selectively reduced before the 3‐carbonyl (100 % regioselectivity); iii) reduction of the 17‐keto group produces epitestosterone almost exclusively (>95 %); that is, the 17α‐OH isomer is selectively formed, as in the case of estrone and epiandrosterone (excellent diastereoselectivity); and iv) despite the eventual reduction of both carbonyl groups taking place at long reaction times, it is possible to minimize the formation of diols by proper selection of the reaction conditions, allowing very high A4 conversions (70–80 %) while keeping very good selectivity (>80 % to monohydroxylated compounds).

## Conclusion

Herein, we have shown that MOF‐808 is a very interesting heterogeneous catalyst for the selective MPV reduction of ketosteroids to the corresponding hydroxysteroids. On the one hand, the particular structure of the Zr_6_ clusters in MOF‐808 (after activation and removal of the capping formate anions) provides coordination vacancies for substrate binding and transformation. On the other hand, the wide pore structure of MOF‐808 compared to the steroid dimensions ensures a good diffusion of the reaction substrates and products to and from the Zr^4+^ active sites. Consequently, MOF‐808 is a highly active catalyst for MPV reduction, requiring the simultaneous coordination of the carbonyl substrate and the secondary alcohol used as a hydride source.

Interestingly, reduction of 17‐ketosteroids produces the corresponding 17α‐OH compound with very high diastereoselectivity, whereas most existing biotransformation methods and chemical routes yield the 17β‐OH isomer as the main product. Therefore, MOF‐808 provides a very attractive catalytic route for the preparation of these challenging compounds, with several improvements compared to alternative bio‐ and chemical processes. We have demonstrated the excellent diastereoselectivity of MOF‐808 for the synthesis of three different 17α‐hydroxysteroids; namely, 17α‐estradiol, androstan‐3β,17α‐diol, and epitestosterone.

Temperature‐dependent kinetic analysis was used to calculate reaction rate constants, apparent activation energies and the thermodynamic parameters of estrone reduction using either *i*PrOH or 2‐BuOH. A close inspection of the energy differences between the two transition states leading to α‐E2 and β‐E2 revealed a major contribution from the entropic term in the case of 2‐BuOH, whereas, in the case of *i*PrOH, the entropic contribution partially neutralizes the enthalpic gain, thus lowering the energy differences between both transitions states. These findings strongly suggest that the diastereoselectivity to α‐E2 originates from the confinement of the reaction inside the MOF cavities, in which the Zr^4+^ active sites are located. This cavity confinement inside the MOF drives the reaction preferentially through the α‐E2 transition state in both *i*PrOH and 2‐BuOH, but this preference is higher with 2‐BuOH, in line with the direct participation of the secondary alcohol in the six‐membered cyclic transition state.

Finally, MPV reduction of androstenedione, containing a C=C bond and two keto groups, has been used to show the excellent chemo‐, regio‐ and diastereoselectivity of the reaction catalyzed by MOF‐808.

## Experimental Section

### Catalyst preparation

*MOF‐808*: MOF‐808 was prepared with slight modifications from an earlier reported procedure by Furukawa et al.^[13]^ Briefly, a solution was prepared with 242.5 mg of ZrOCl_2 ⋅_ 8H_2_0 (0.75 mmol), 105 mg of trimesic acid (0.5 mmol) and 22.5 mL of a DMF/HCO_2_H (1 : 1, *v*/*v*) mixture. The solution was transferred into a Teflon lined autoclave and heated inside an oven at 130 °C for 48 h. After cooling down to room temperature, the material was recovered by centrifugation and washed for 3 days with DMF (changing the solvent 2 times per day) and for another 3 days with EtOH (changing the solvent twice per day). After removing the solvent by centrifugation, the solid was dried in air. X‐ray diffraction (PhillipsX'Pert, Cu_Kα_ radiation) was used to confirm the expected structure type and high crystallinity of the material. According to N_2_‐adsorption experiments, calculated specific surface area (*S*_BET_) and pore volume were 1345 m^2^ g^−1^ and 0.60 cm^3^g^−1^, respectively. A summary of the most relevant characterization data of this material is shown in Figure S1.

*Other catalysts*: The Supporting Information contains details of the preparation of other noncommercial catalysts used in this work for comparison purposes.

### Catalytic experiments

*MPV reduction of ketosteroids*: Particular reaction conditions for each substrate are indicated in the text. To illustrate the typical procedure used, reduction of estrone over MOF‐808 with *i*PrOH will be described. Estrone (20 mg, 0.08 mmols) was dissolved in *i*PrOH (1 mL, ca. 16 equiv.) and introduced inside a screw cap glass reactor containing MOF‐808 (5 mg, ca. 18 mol% Zr referred to estrone) and kept at the selected reaction temperature under magnetic stirring (stirring rate was kept>600 rpm to ensure optimal mixing and to avoid diffusional limitations). Time‐evolution of products was analyzed by GC (Agilent 7890A, equipped with a FID detector and a DB5 30 m×0,25 mm×0,25 μm column) on sample aliquots taken at fixed time intervals. Product identification was done by GC‐MS and by comparison with pure standards. Peak separation of the different diastereoisomers in the resulting chromatograms was good enough to extract the concentration of the individual species. More accurate diastereomeric ratios were obtained at the end of the reaction from the corresponding ^1^H NMR spectra, as detailed in the Supporting Information.

*Estrone reduction with NaBH_4_*: A solution was prepared by dissolving estrone (40 mg, 0.15 mmol) in ethanol (5 mL). A second ethanolic solution (1 mL) containing NaBH_4_ (18.9 mg) was added to the first one at 353 K under stirring and kept at this temperature for 15 min. Then, the temperature was raised to reflux, and the heating was stopped just after the boiling started, and let it stand for 5 min. To stop the reaction, 10 mL of H_2_O was added and the mixture was cooled in a water‐ice bath for 15 min, whereupon a white precipitate of the product formed. Finally, the product was recovered by filtration and washed with cold water and analyzed by GC as described above.

## Conflict of interest

The authors declare no conflict of interest.

## Supporting information

As a service to our authors and readers, this journal provides supporting information supplied by the authors. Such materials are peer reviewed and may be re‐organized for online delivery, but are not copy‐edited or typeset. Technical support issues arising from supporting information (other than missing files) should be addressed to the authors.

Supporting Information
