# Supplementary material for: One‐Step Chemo‐, Regio‐ and Stereoselective Reduction of Ketosteroids to Hydroxysteroids over Zr‐Containing MOF‐808 Metal‐Organic Frameworks
Source: Chemistry. 2021 Jun 8;27(41):10766–75. doi: 10.1002/chem.202100967 (PMC8362071; doi:10.1002/chem.202100967)
Supplement: Supplementary file 1 — Supporting Information [file CHEM-27-10766-s001.pdf]

Chemistry–A European Journal

Supporting Information

One-Step Chemo-, Regio- and Stereoselective Reduction of Ketosteroids to Hydroxysteroids over Zr-Containing MOF-808 Metal-Organic Frameworks

H.-H. Mautschke and F. X. Llabrés i Xamena*

CONTENTS

A1) Structure and characterization of MOF-808 2

A2) Synthesis of other non-commercial catalysts used in the work............................3

A3) NMR identification of reaction products 4

A4) Kinetic analysis of E1 reduction 9

A5) Reduction of androstenedione (A4) 12

A1) Structure and characterization of MOF-808

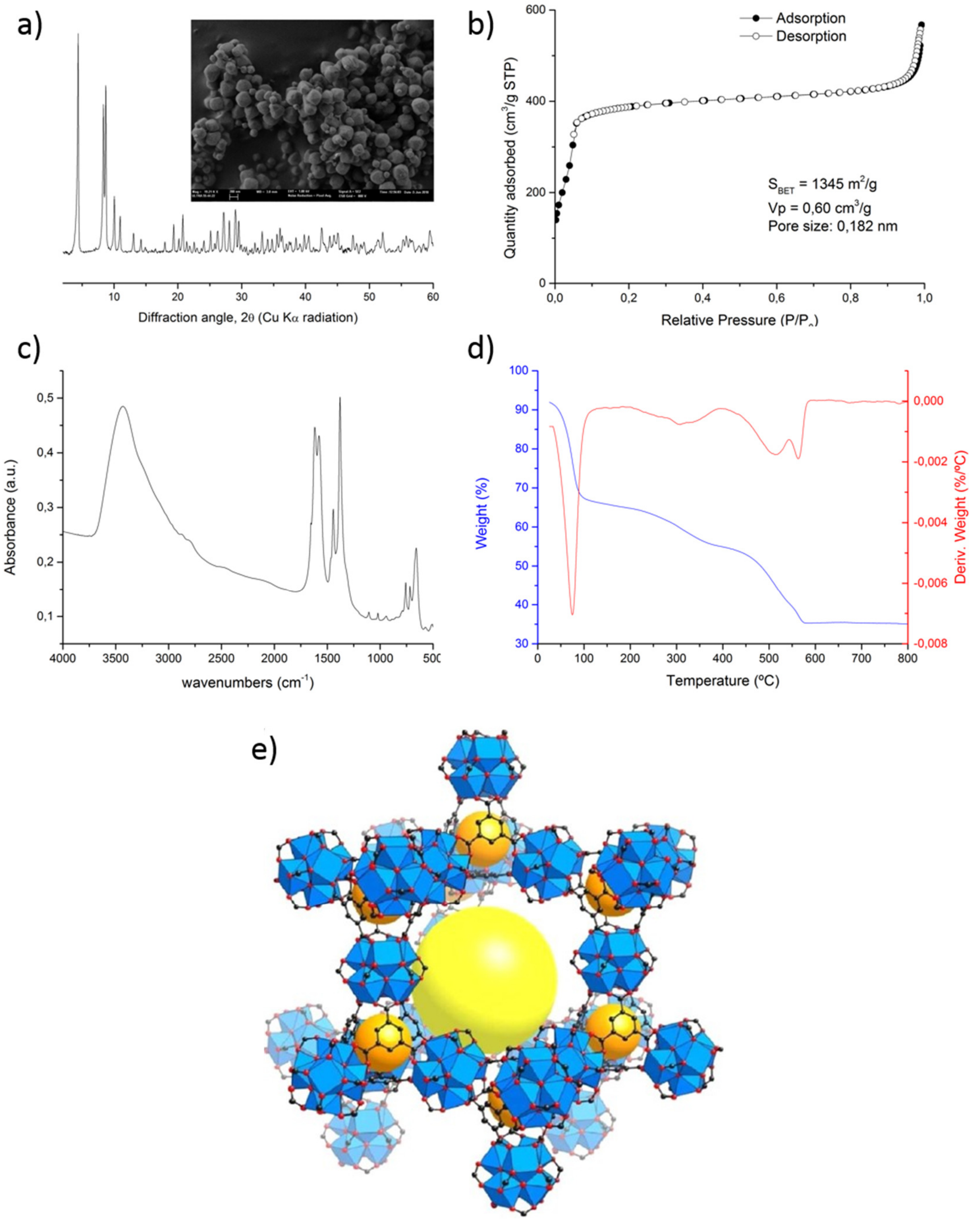

Figure S1. Characterization of MOF-808. a) XRD powder diffraction pattern (Cu Kα radiation). The inset shows the corresponding FESEM image, in which the crystal size (*ca.* 200 nm) and the octahedral-shaped morphology is evidenced. b) N_2 adsorption/desorption isotherm (at 77K) and corresponding textural parameters. c) FTIR spectrum. The absorption bands observed are analogous to those previously described for MOF-808 (see for instance: J. Xu et al., *New J. Chem.* **2019**, *43*, 4092-4099). d) TGA and DTG curves. e) Structure of MOF-808. Reproduced with permission from Furukawa et al. *J. Am. Chem. Soc.* **2014**, *136*, 4369–4381. Copyright 2014 American Chemical Society.

A2) Synthesis of other non-commercial catalysts used in the work

Zr-beta. Preparation of this catalyst consisted of two steps:

a) Synthesis of dealuminated Zr-beta seeds

1.85 g of $AlCl_3 \cdot 6H_2O$ were dissolved in 4.33 g of water. To this solution, 45.2 g of tetraethylammonium hydroxide solution (TEAOH, 35 wt. % in H_2O) were added, followed by 40.0 g of tetraethyl orthosilicate (TEOS), and the mixture was stirred until evaporation of the ethanol formed by hydrolysis of TEOS. The final composition of the gel was:

SiO_2: 0.56 TEAOH: 0.02 Al_2O_3: 6.5 H_2O

The gel was then transferred into a teflon lined autoclave and heated to 140ºC for 3 days with stirring. The solid product was recovered by filtration, washed with distilled water and dried at 100ºC. The resulting zeolite was dealuminated by treating 1 g of the solid with 60 g of HNO_3 (60 wt%) at 80ºC for 24 h. The dealuminated solid was filtered, washed with water and dried at 100ºC. The final Si/Al ratio of the solid was higher than 2000, as determined by elemental analysis.

b) Synthesis of aluminum-free Zr-beta zeolite

30 g of TEOS and 33.0 gr of TEAOH (35 wt. % in H_2O) were placed inside a teflon lined autoclave. To this, a solution containing 0.39 g of $ZrOCl_2 \cdot 8H_2O$ in 2.75 g water was added, and the mixture was stirred until evaporation of the ethanol formed by hydrolysis of TEOS. To this solution, 3.27 g of HF (48 wt%) were added, and the resulting viscous gel was mixed with a aqueous suspension of 0.36 g of preformed dealuminated Zr-beta seeds in 2 g of water. The final composition of the gel was:

SiO_2: 0.54 TEAOH: 0.008 ZrO_2: 0.54 HF: 7.5 H_2O

The gel was then heated to 140ºC for 14 days with stirring. The solid product was recovered by filtration, washed with distilled water and dried at 100ºC. The final Si/Zr ratio of the solid was 148, as determined by elemental analysis.

A3) NMR identification of reaction products

Estrone (E1) reduction to 17α- and 17β-estradiol (α-E2 and β-E2)

^{1}H NMR spectra of E1, α-E2 and β-E2 are shown in Figure S2, together with the spectrum of a reaction crude obtained at almost full conversion of E1. The complete list of peaks of the pure compounds is given below. The most characteristics peaks correspond to the protons of the group 18-CH_3 (labelled **a** in the figure), at 0.82, 0.61 and 0.66 ppm for E1, α-E2 and β-E2, respectively. Additionally, both α-E2 and β-E2 show signals corresponding to 17-H (labelled **b**) and the 17-OH group (labelled **c**), which are absent in the spectrum of E1. Diastereomeric ratio α-E2:β-E2 can be accurately determined from the intensity ratio of peaks at 0.61/0.66 (**a**) and 4.33/4.47 (**c**), for α-E2 and β-E2 respectively. Signals **b** have not been used, due to strong peak overlap.

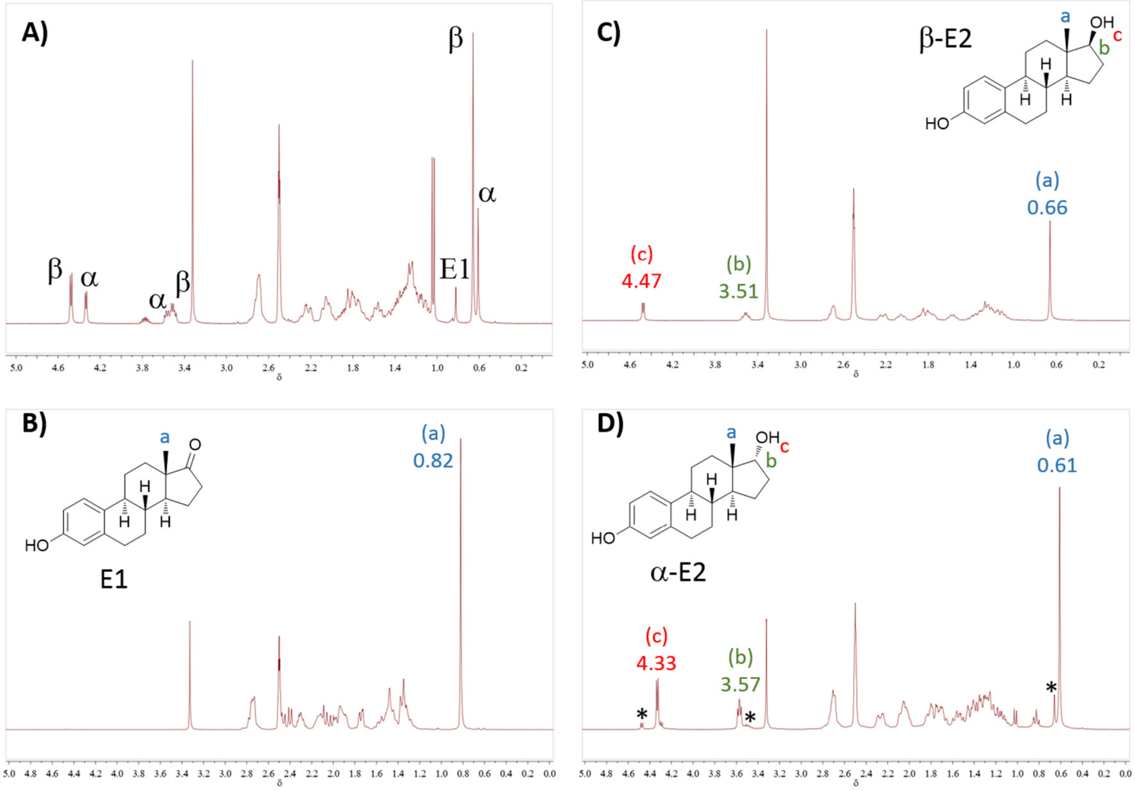

Figure S2. ^{1}H NMR spectra of: (A) the reaction crude obtained at almost full estrone conversion (some estrone still left, marked with E1); and reference spectra of (B) estrone, (C) 17-β-estradiol (β-E2), and (D) 17-α-estradiol (α-E2). The most prominent peaks of each compound have been indicated. In (D), the asterisks mark some traces of β-E2 detected together with α-E2.

α-E2: ^{1}H NMR (300 MHz, DMSO-d_6): δ 8.96 (s, 1H), 7.05 (d, J = 8.4 Hz, 1H), 6.50 (dd, J = 8.4, 2.5 Hz, 1H), 6.43 (d, J = 2.3 Hz, 1H), 4.33 (d, J = 4.2 Hz, 1H), 3.61 – 3.54 (m, 1H), 2.71 (s, 2H), 2.27 (d, J = 10.1 Hz, 1H), 2.13 – 1.96 (m, 2H), 1.82 (d, J = 9.6 Hz, 1H), 1.77 – 1.62 (m, 2H), 1.61 – 1.49 (m, 1H), 1.49 – 1.05 (m, 6H), 0.61 (s, 3H).

β-E2: ^{1}H NMR (300 MHz, DMSO-d_6): δ 8.96 (s, 1H), 7.04 (d, J = 8.4 Hz, 1H), 6.50 (dd, J = 8.3, 2.4 Hz, 1H), 6.43 (s, 1H), 4.47 (d, J = 4.8 Hz, 1H), 3.51 (dd, J = 13.0, 8.2 Hz, 1H), 2.69 (s, 2H), 2.22 (d, J = 12.6 Hz, 1H), 2.06 (s, 1H), 1.85 (t, J = 12.7 Hz, 3H), 1.57 (d, J = 9.1 Hz, 1H), 1.46 – 0.98 (m, 7H), 0.66 (s, 3H).

Epiandrosterone (EPIA) reduction to 5α-androstan-3β,17α-diol (α-EPIAdiol) and 5α-androstan-3β,17β-diol (β-EPIAdiol)

Analogous to the case of E1 reduction described above, the procedure used for the quantification of products coming from the reduction of EPIA and corresponding diastereomeric ratio were based on the analysis of the ^{1}H NMR spectra of the reaction crude and comparison with the pure substances. The most characteristic signals of the two androstandiols and the experimental spectra obtained are shown below:

α-EPIAdiol: ^{1}H NMR (300 MHz, $CDCl_3$): δ 3.72 (dd, J = 5.7, 3.6 Hz, 1H), 3.60 (m, 1H), 0.82 (s, 3H), 0.65 (s, 3H).

β-EPIAdiol: ^{1}H NMR (300 MHz, $CDCl_3$): δ 3.60 (m, 2H), 0.82 (s, 3H), 0.73 (s, 3H).

Note that the protons of the 18-CH_3 group (labelled **a** in Figure S3) have different chemical shifts for α-EPIAdiol and β-EPIAdiol: 0.65 and 0.73 ppm, respectively. Furthermore, the two equatorial protons 3-H and 17-H have an identical chemical shifts in β-EPIAdiol (signals **c** and **d** at 3.60 ppm), but they appear as two separate signals in α-EPIAdiol (**c** and **d**), having the equatorial 17-H a slight downfield shift (3.72 ppm). These characteristics have been previously described in the literature[1] and were therefore used for the characterization of products. EPIA can be also unambiguously identified from the ^{1}H-NMR spectra, as both CH_3 groups (18-CH_3 and 19-CH_3,labelled **a** and **b** in Figure S3) appear at have a downfield chemical shift compared to diols, α-EPIAdiol and β-EPIAdiol: 0.84 and 0.86 ppm.

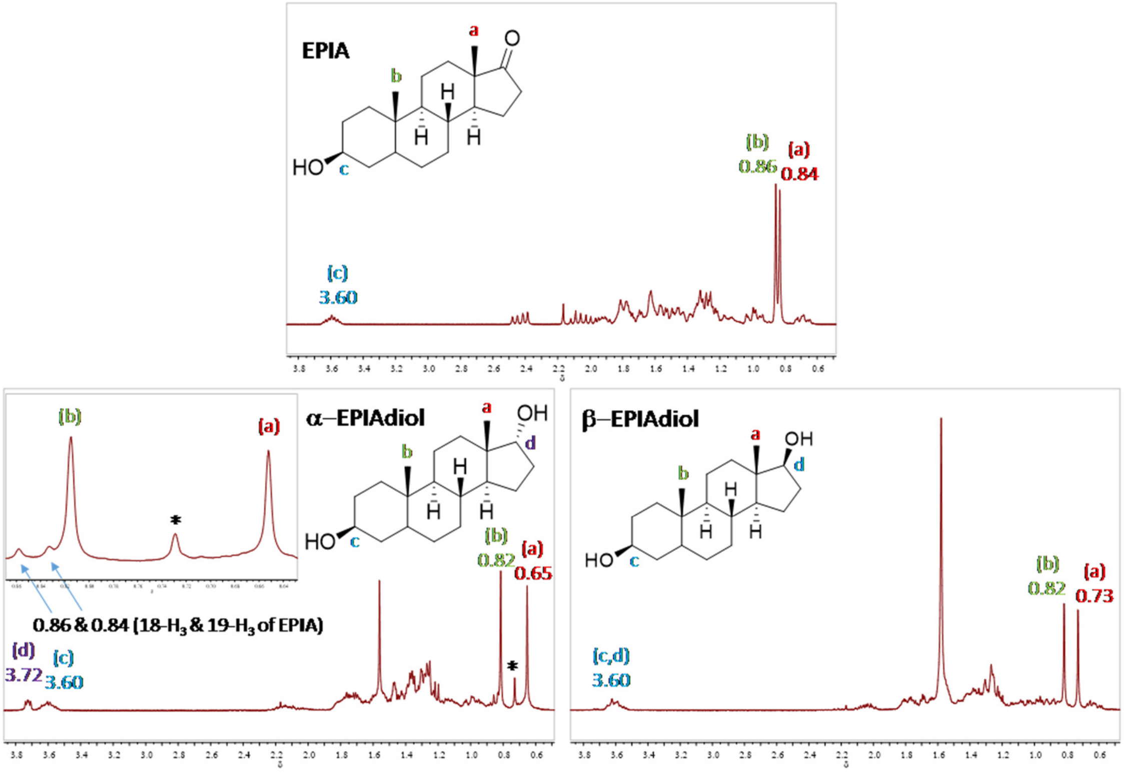

Figure S3. ^{1}H NMR spectra of: (*top*) EPIA; (*bottom left*) α-EPIAdiol, with small traces of EPIA (at 0.86 and 0.84 ppm, indicated by the arrows in inset), and β-EPIAdiol (at 0.73 ppm, marked with an asterisk); and (*bottom right*) β-EPIAdiol.

Reduction of Δ^{4}-androstene-3,7-dione (androstenedione, A4)

Given the complexity of the molecule and products formed, it was necessary to complement ^{1}H and ^{13}C-NMR measurements for the complete characterization of the reaction products. Most relevant signals of the products are as follows:

E: ^{1}H NMR (300 MHz, DMSO-d$_6$): δ 4.30 (d, J = 4.1 Hz, 1H), 3.52 (dd, J = 10.7, 6.3 Hz, 1H), 1.14 (s, 3H), 0.63 (s, 3H); **^{13}C NMR** (300 MHz, DMSO-d$_6$): δ 198.05 (1C), 77.75 (1C).

T: ^{1}H NMR (300 MHz, DMSO-d$_6$): δ 4.46 (d, J = 4.8 Hz, 1H), 3.43 (dd, J = 8.4, 4.9 Hz, 1H), 1.14 (s, 3H), 0.68 (s, 3H); **^{13}C NMR** (300 MHz, DMSO-d$_6$): δ 198.01 (1C), 79.86 (1C).

Product E was first isolated from the reaction crude at complete conversion of A4 and purified by TLC (PE/EtOAc, 4/6). This product was used as reference for the ensuing NMR studies (spectrum labelled E in Figure S4. The ^{1}H NMR spectrum of the purified

fraction showed some signals corresponding to residual solvent, but they do not interfere with the relevant signals of E).

In the ^{1}H-NMR spectra of products E and T, a clear upfield chemical shift of the signal corresponding to the 18-CH_3 group is observed for E (**a**, 0.63 ppm) with respect to T (0.68 ppm). Further changes are observed in the signals of 17-H and 17-OH (**c** and **d**), undergoing downfield and upfield chemical shifts: 3.52/3.43 and 4.30/4.46 ppm for E and T, respectively. By carefully analyzing the ^{1}H NMR spectrum of the crude reaction (with signals at 0.63, 3.52 and 4.30 ppm), it is evident that the keto group in 17-position is reduced with very high selectivity towards the 17α isomer (E).

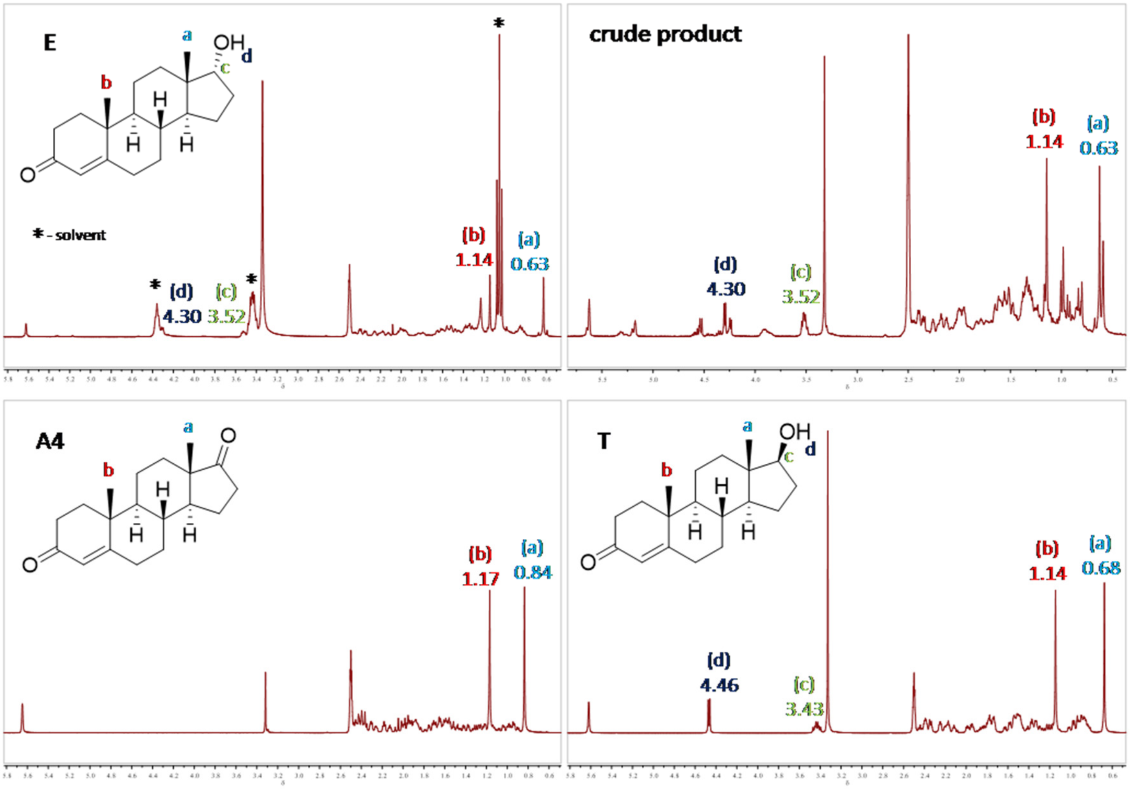

Figure S4. ^{1}H-NMR of (*top left*) epitestosterone, E (purified from the reaction crude by TLC). Peaks marked with an asterisk correspond to residual solvent; (*top right*) crude product after removal of the catalyst by centrifugation; (*bottom left*) starting compound, androstenedione, A4; and (*bottom right*) a reference sample of testosterone, T.

Moreover, by comparing the ^{13}C-NMR spectra of the reaction crude and the starting A4 product shown in Figure S5, a large upfield chemical shift of the 17-C=O signal **a** is observed, from 219.36 ppm for A4 to 77.75 ppm, due to reduction on C-17 from 17-C=O to 17-C-OH. Note that this new signal of 17-C-OH coincides again with that of E, while a dowfield shifted signal is observed for pure T: 79.86 ppm. Therefore, also the ^{13}C NMR spectra confirm that E is formed selectively over T upon reduction of A4. After a careful

analysis of the areas of both ^{1}H and ^{13}C NMR signals, the E:T diastereomeric ratio of the final product is estimated to be higher than 95%. Meanwhile, the signal **b** corresponding to the 13-C=O groups is still well visible in the reaction crude, though slightly shifted with respect to A4: from 197.9 to 198.05 ppm. This clearly demonstrates that reduction at position 17 takes place prior to the reduction at position 3.

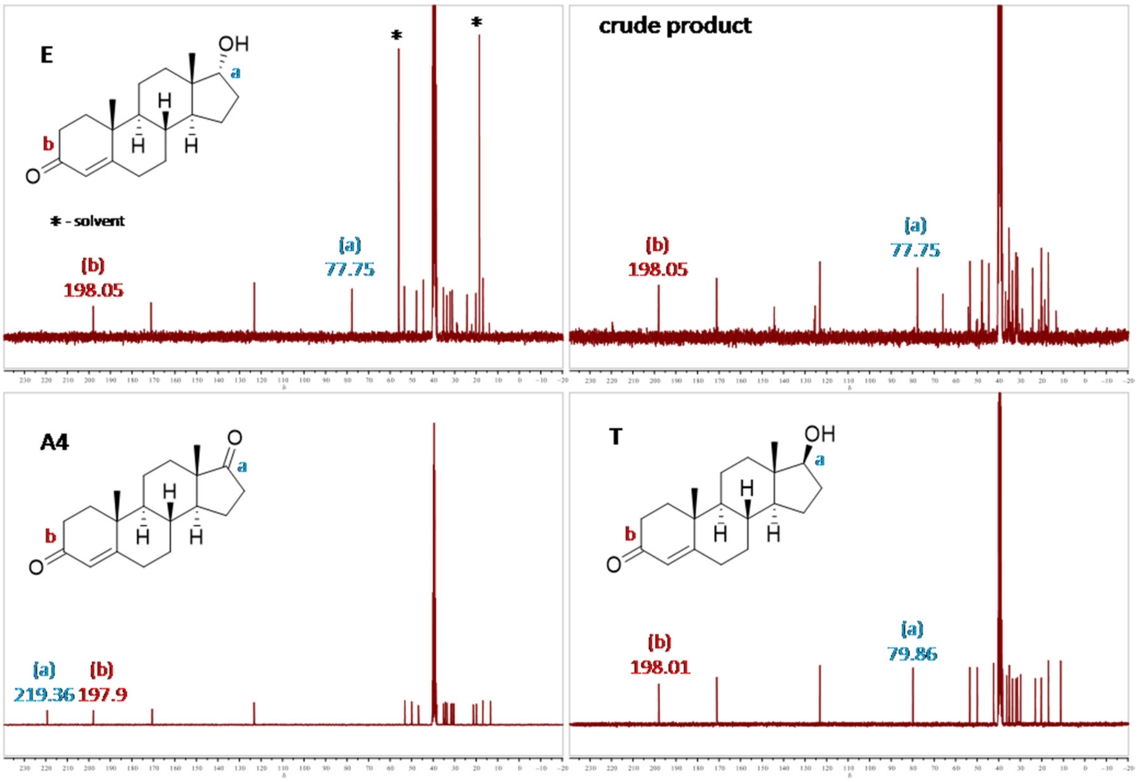

Figure S5. ^{13}C-NMR of (*top left*) epitestosterone, E (purified from the reaction crude by TLC). Peaks marked with an asterisk correspond to residual solvent; (*top right*) crude product after removal of the catalyst by centrifugation; (*bottom left*) starting compound, androstenedione, A4; and (*bottom right*) a reference sample of testosterone, T. ,In the purified fraction of E (left, top).

A4) Kinetic analysis of E1 reduction

For the calculation of the kinetic parameters of MPV reduction of estrone (E1) to 17α- and 17β-estradiol (α-E2 and β-E2), the following reaction scheme was considered:

$$E1 \rightarrow \alpha - E2 + \beta - E2 \quad \text{(Equation 1)}$$

We assumed that interconversion of products, α-E2 and β-E2, as well as the reversed reaction of products to the corresponding starting compound can be neglected. Furthermore, the reducing agent (e.g., iPrOH, 2-BuOH, …) was used in high excess (16 eq.), so the reaction was carried out under pseudo-first order conditions. Following this approach, the overall rate of E1 conversion, k, is given by the sum of the formation products of α-E2 and β-E2. Thus,

$$k = k_\alpha + k_\beta \quad \text{(Equation 2)}$$

Since there is no interconversion between α-E2 and β-E2, the ratio α-E2/β-E2 is maintained constant throughout the whole reaction at a given temperature, and it is simple given by:

$$\frac{[\alpha - E2]}{[\beta - E2]} = \frac{k_\alpha}{k_\beta} = const \quad \text{(Equation 3)}$$

Our experimental data of α-E2 and β-E2 yields with reaction time confirmed that the ratio between these two species was indeed constant and independent of the level of E1 conversion, which proves the validity of the above assumption that no interconversion occurs between reaction products. The reaction thus follows the typical Curtin-Hammet/Weinstein-Holness conditions.[2]

With the above considerations, the rate equation can be formulated as:

$$-d[E1]/dt = k\,[E1] = k_\alpha[E1] + k_\beta[E1] = (k_\alpha + k_\beta)[E1] \quad \text{(Equation 4)}$$

or in its integrated form:

$$\ln[E1] = ln[E1]_0 - k * t \quad \text{(Equation 5)}$$

where $[E1]_0$ is the initial concentration of E1 at t = 0. Therefore, the overall reaction rate constant, k, is obtained from the slope of the linear plot of ln[E1] against reaction time, t. The good linearity observed in all cases demonstrates again the validity of the adopted model. Finally, individual rate constants k_α and k_β, are obtained by combining Equations 2 and 3.

Thermodynamic calculations

Arrhenius plots. Apparent activation energies of the reaction, E_a, and pre-exponential factors, k_0, for both α-E2 and β-E2 were calculated from the corresponding Arrhenius plots shown in Figure 3 of the main text, by applying the well-known Arrhenius equation:

$$k(T) = k_0 * e^{-E_a/RT} \quad \text{(Equation 6)}$$

which in its logarithmic form can be written as:

$$\ln(k(T)) = \ln(k_0) - E_a/RT \quad \text{(Equation 7)}$$

Using the obtained values of E_a and k_0, the theoretical evolution of k_α and k_β with the temperature can be calculated by applying Equation 6. The goodness of the fit is evidenced by comparing these theoretical curves and the experimental constants determined from the kinetic analysis, as shown in Figure S6 for the reactions performed in 2-BuOH.

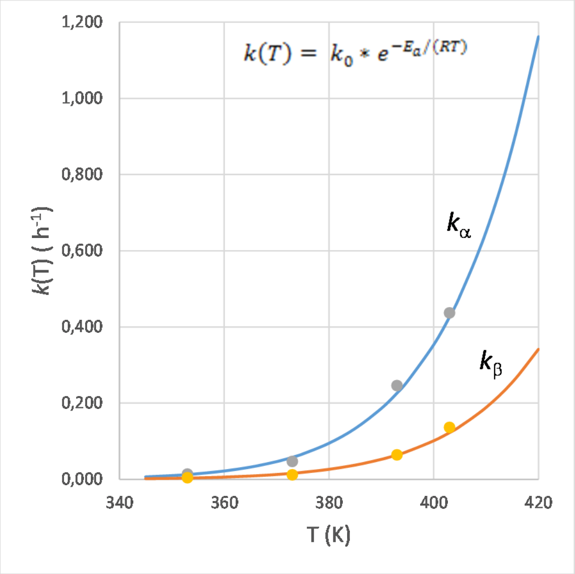

Figure S6. Calculated evolution with temperature of rates of α-E2 and β-E2 formation (solid curves) and experimental values of k_α and k_β determined form the kinetic data analysis (discrete points, corresponding to 353, 373, 393 and 403 K).

Eyring-Polanyi plots. Following the Transition State Theory, the Eyring-Polanyi equation can be used to calculated the Gibbs free energy of activation ($\Delta G^\ddagger$), enthalpy of activation ($\Delta H^\ddagger$) and entropy of activation ($\Delta S^\ddagger$). The Eyring-Polanyi equation is given by Equations 8 and 9:

$$k(T) = \frac{k_B\, T}{h} e^{-\Delta G^\ddagger/RT} \quad \text{(Equation 8)}$$

$$\ln\left(\frac{k}{T}\right) = \frac{-\Delta H^\ddagger}{RT} + \ln\left(\frac{k_B}{h}\right) + \frac{\Delta S^\ddagger}{R} \quad \text{(Equation 9)}$$

where k_B is the Boltzmann constant ($1.38064852 \cdot 10^{-23}$ J·K^{-1}); h is the Planck constant ($6.626070150 \cdot 10^{-34}$ J·s) and R is the gas constant (8.314 J K^{-1} mol^{-1}). Then, the values of $\Delta H^{\ddagger}$ and $\Delta S^{\ddagger}$ can be obtained by plotting $\ln\left(\frac{k}{T}\right)$ versus T.

From the above equation, the free Gibbs free energy of activation ($\Delta G^{\ddagger}$) of the reaction can be calculated by:

$$\Delta G^{\ddagger} = RTln\left(\frac{k_B T}{h}\right) - RTln(k) \qquad \text{(Equation 10)}$$

or simply by applying the well-known Gibbs Equation 11 to the calculated values of $\Delta H^{\ddagger}$ and ($\Delta S^{\ddagger}$):

$$\Delta G^{\ddagger} = \Delta H^{\ddagger} - T\Delta S^{\ddagger} \qquad \text{(Equation 11)}$$

We want to recall here that, despite the similarities between the Arrhenius and the Eyring-Polany equations (compare Equations 6 and 8) , the terms activation energy, E_a, and Gibbs free energy, $\Delta G^{\ddagger}$, are not equivalent. While $\Delta G^{\ddagger}$ contains both enthalpic and entropic terms, in the Arrhenius equation the entropic term is included in the pre-exponential factor, k_0. For elementary reactions, approximate relationships between both equations are given by Equations 12 and 13:

$$E_a = \Delta H^{\ddagger} + RT \qquad \text{(Equation 12)}$$

$$k_0 = \frac{k_B T}{h} e^{1+\Delta S^{\ddagger}/R} \qquad \text{(Equation 13)}$$

The Eyring-Polanyi plots obtained for the reduction of E1 to α-E2 and β-E2 over MOF-808 using either iPrOH or 2-BuOH as reducing agent are shown in Figure S7, in which the calculated values of $\Delta G^{\ddagger}$, $\Delta H^{\ddagger}$ and $\Delta S^{\ddagger}$ are also shown.

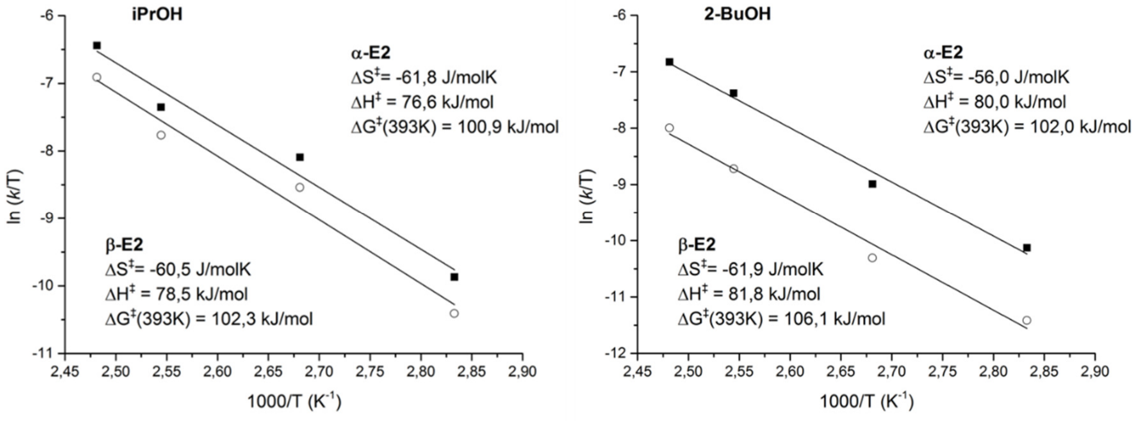

Figure S7. Eyring-Polanyi plots and calculated $\Delta G^{\ddagger}$, $\Delta H^{\ddagger}$ and $\Delta S^{\ddagger}$ parameters for the E1 reduction over MOF-808 in iPrOH (*left*) and 2-BuOH (*right*).

A5) Reduction of androstenedione (A4)

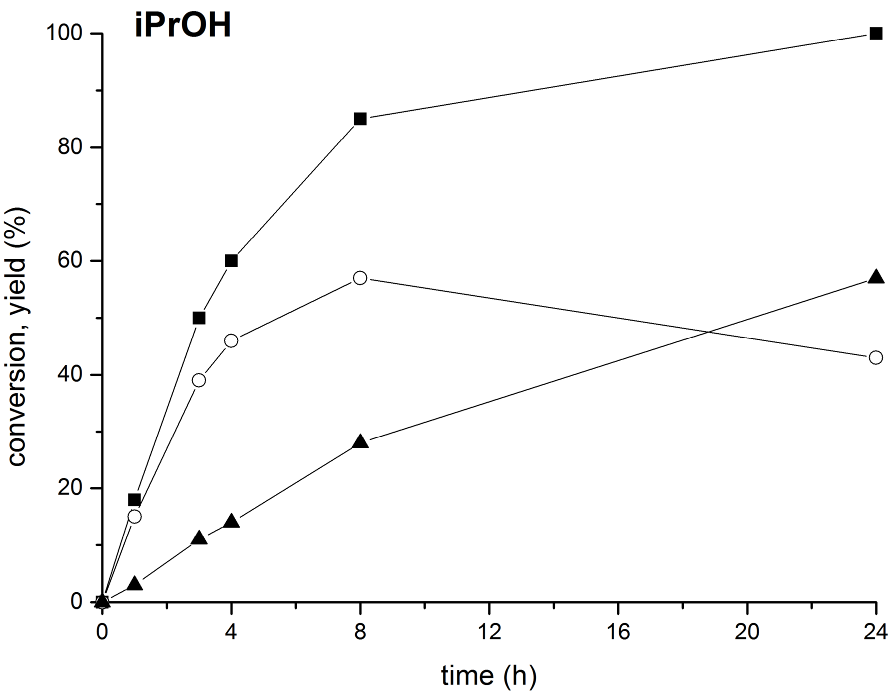

Figure S8. (-■-) Androstenedione conversion; (-○-) yield of mono-hydroxylated (testsosterone+epitestosterone); and (-▲-) yield of dihydroxylated products obtained over MOF-808 with iPrOH at 353 K.

Table S1. Catalytic data obtained for the reduction of A4 with 2-BuOH over MOF-808 in the 333-393 K range.

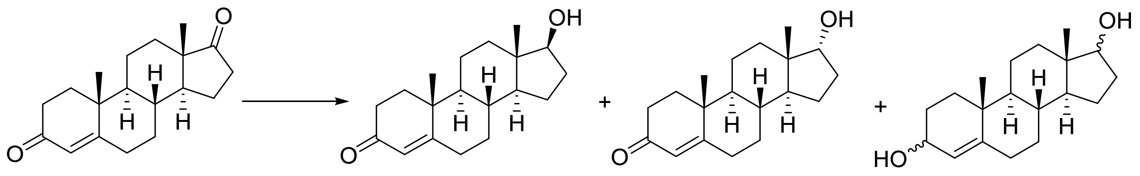

A4 **T** **ET** **Diol isomers**

				Selectivity (mol%)[a]			
				Monohydroxylated compounds			Double reduction
Temperature (K)	Time (h)	Conversion **A4** (mol%)		**ET**	**T**	**ET+T**	**Diols**
333	8	20		81	4	85	15
	24	64		77	4	81	19
	48	86		73	4	77	23
	72	95		64	3	67	33
353	1	12		81	4	85	15
	3	48		77	3	81	19
	4	59		74	4	78	22
	8	89		62	3	65	35
	24	99		50	2	52	48
393	0.5	40		78	4	82	18
	0.75	70		67	3	70	30
	1	92		48	2	50	50
	3	99		38	2	40	60

[a] Product distribution determined from ^{1}H and ^{13}C NMR spectra, as detailed above. The amount of T formed was very low, close to the detection limits of the technique (< 5% with respect to ET). Given the low yield of T, the numbers given in the Table are only approximate, and the actual amount of T is probably lower. No additional products were detected, and the mass balance at the end of the reaction was > 98% in all cases.

References

[1] A. C. Hunter, C. Collins, H. T. Dodd, C. Dedi, S. J. Koussoroplis, *J. Steroid Biochem. Mol. Biol.* **2010**, *122*, 352–358.

[2] J. I. Seeman, *Chem. Rev.* **2002**, *83*, 83–134.
